# Toxicity and feasibility of adjuvant high-dose interferon alpha-2b in patients with melanoma in clinical oncologic practice

**DOI:** 10.1038/sj.bjc.6690595

**Published:** 1999-08

**Authors:** A Ravaud, C Bedane, L Geoffrois, T Lesimple, M Delaunay

**Affiliations:** Department of Medicine, Institut Bergonié, 180 rue de Saint-Genès, Bordeaux Cedex, 33076, France; Department of Dermatology, CHU Dupuytren, Limoges, France; Department of Medicine, Centre Alexis Vautrin, Comprehensive Cancer Center, Nancy, France; Department of Medicine, Centre Eugène Marquis, Comprehensive Cancer Center, Rennes, France; Department of Oncodermatology, CHU Pellegrin, Bordeaux, France

**Keywords:** melanoma, immunotherapy, adjuvant treatment, interferon alpha, toxicity

## Abstract

To assess the feasibility and toxicity profile of high-dose interferon alpha-2b (IFN-α-2b) in the adjuvant treatment of patients with cutaneous malignant melanoma outside the reference ECOG 1684 clinical trial, we conducted a prospective follow-up in an identical population of patients (cutaneous melanoma, T4 and/or N1) treated by intravenous IFN-α-2b:20 MIU m^−2^, 5 days a week for 4 weeks; and subcutaneous:10 MIU m^−2^, 3 times a week for 11 months. Thirty-six consecutive patients were considered in four different institutions. The frequency and severity of side-effects related to IFN-α, as well as the percentage of the planned dose given to patients, were identical to those reported in the initial report by ECOG. Fifty per cent and 47% of patients had a grade 3/4 WHO toxicity in the induction and consolidation phase respectively. A dose modification was necessary for 47.2% and 55.8% of the patients in the induction and consolidation phase respectively. The schedule and dose of high-dose IFN-α-2b in the adjuvant treatment of cutaneous malignant melanoma, as reported by ECOG 1684, is feasible. The significant toxicity reported in ECOG 1684 was also seen in our patients. Nevertheless, this protocol will not be a ‘standard’ treatment until the publication of the ECOG 1690 trial. © 1999 Cancer Research Campaign


*British Journal of Cancer* (1999) **80**(11), 1767–1769
© 1999 Cancer Research Campaign
Article no. bjoc.1998.0595

### Toxicity and feasibility of adjuvant high-dose interferon alpha-2b in patients with melanoma in clinical oncologic practice

**A Ravaud[1], C Bedane[2], L Geoffrois[3], T Lesimple[4] and M Delaunay[1,5]**

[1]Department of Medicine, Institut Bergonié, Comprehensive Cancer Center, 180 rue de Saint-Genès 33076, Bordeaux Cedex, France; [2]Department of Dermatology, CHU Dupuytren, Limoges, France; [3]Department of Medicine, Centre Alexis Vautrin, Comprehensive Cancer Center, Nancy, France; [4]Department of Medicine, Centre Eugène Marquis, Comprehensive Cancer Center, Rennes, France; [5]Department of Oncodermatology, CHU Pellegrin, Bordeaux, France

**Summary** To assess the feasibility and toxicity profile of high-dose interferon alpha-2b (IFN-α-2b) in the adjuvant treatment of patients with cutaneous malignant melanoma outside the reference ECOG 1684 clinical trial, we conducted a prospective follow-up in an identical population of patients (cutaneous melanoma, T4 and/or N1) treated by intravenous IFN-α-2b:20 MIU $m^{-2}$, 5 days a week for 4 weeks; and subcutaneous:10 MIU $m^{-2}$, 3 times a week for 11 months. Thirty-six consecutive patients were considered in four different institutions. The frequency and severity of side-effects related to IFN-α, as well as the percentage of the planned dose given to patients, were identical to those reported in the initial report by ECOG. Fifty per cent and 47% of patients had a grade 3/4 WHO toxicity in the induction and consolidation phase respectively. A dose modification was necessary for 47.2% and 55.8% of the patients in the induction and consolidation phase respectively. The schedule and dose of high-dose IFN-α-2b in the adjuvant treatment of cutaneous malignant melanoma, as reported by ECOG 1684, is feasible. The significant toxicity reported in ECOG 1684 was also seen in our patients. Nevertheless, this protocol will not be a 'standard' treatment until the publication of the ECOG 1690 trial.

**Keywords:** melanoma; immunotherapy; adjuvant treatment; interferon alpha; toxicity

In January 1996, ECOG (Eastern Cooperative Oncology Group) published the first prospective clinical trial (ECOG 1684) indicating a significant increase in survival for patients with T4 or N1 melanoma treated with adjuvant high-dose interferon alpha-2b (IFN-α-2b; Kirkwood et al, 1996). Nevertheless, this treatment was associated with moderate to severe side-effects for a long time during and after treatment (Cole et al, 1996; Kirkwood et al, 1996). Therefore, apart from the issue of the indication for such a treatment, and while waiting for the results of the forthcoming trial ECOG 1690, we planned to treat patients with T4 or N1 melanoma who were referred to our institutions with the identical ECOG 1684 treatment, and to evaluate prospectively the tolerance and feasibility of this treatment outside a clinical trial.

### MATERIALS AND METHODS

To be included, patients had to fulfill limited inclusion criteria. Only patients with a cutaneous melanoma classified as T4 or N1 were treated. In the latter group, an extensive regional lymphadenectomy was strongly recommended. The evaluation of other underlying co-morbidity was left to the discretion of the physician.

The planned treatment was a dose of 20 MIU $m^{-2}$ IFN-α-2b given intravenously, 5 days a week for 4 weeks (induction phase) followed by subcutaneous IFN-α-2b at 10 MIU $m^{-2}$, 3 times a week for 11 months (consolidation phase) (Kirkwood et al, 1996). The treatment had to start less than 6 weeks after the definitive surgical treatment.

Toxicity was evaluated using the World Health Organization (WHO) grading criteria. All patients received paracetamol and anti-emetics for the first few days to prevent fever and nausea or vomiting. Thereafter, symptomatic treatment was tailored to toxicity. Haematological and hepatic parameters were checked 1–3 times a week during the induction phase and 1–4 times a month during the consolidation phase. Patients were seen by the physicians weekly in the induction phase and monthly during the consolidation phase. No mandatory dose modification was planned but particular care was recommended for severe psychiatric disorders, grade 3 increase in transaminases and grade 4 neutropenia. Thyroid function was checked every 3–6 months during treatment.

### RESULTS

Thirty-six patients started treatment between March 1996 to December 1997. Thirty-four patients had a N1 disease, of these, 31 had an extensive regional nodal dissection, one patient had a T4 disease and another had been treated for a single metastatic lesion by surgery. The median age was 54, with a range from 8 to 75 years old.

### Induction phase

During the induction phase, 17 patients received 100% and 24 (66.6%) more than 80% of the planned dose (Table 1). Thirteen patients (36.1%) experienced grade 3 toxicity while five patients

*Received 3 June 1998*
*Revised 9 October 1998*
*Accepted 4 November 1998*

*Correspondence to:* A Ravaud

**Table 1** Dose of IFN-α-2b

| Number of patients receiving | 100% | > 80% | (%) |
|---|---|---|---|
| Induction phase | 17/36 | 24/36 | (66.6) |
| Consolidation phase | | | |
| 1–3 months | 23/34 | 24/34 | (70.6) |
| 4–6 months | 17/31 | 21/31 | (67.8) |
| 7–9 months | 13/26 | 13/26 | (50) |
| 10–11 months | 7/17 | 10/17 | (58.8) |

**Table 2** Toxicity by type and degree during induction phase

| WHO grade (*n* = 36) | 1 | 2 | 3 | 4 |
|---|---|---|---|---|
| Asthenia | 11 | 9 | 5 | 1 |
| Fever | 2 | 13 | 1 | – |
| Nausea/vomiting | 12 | 2 | 3 | – |
| Diarrhoea | 2 | – | 1 | – |
| Psychiatric disorders | 4 | 4 | 1 | 1 |
| Headache/myalgia | – | 4 | 1 | – |
| Alopecia | 4 | – | – | – |
| Infectious process | – | 1 | 1 | 1 |
| Neutropenia | 7 | 10 | 9 | 3 |
| Thrombopenia | 2 | – | – | – |
| Hepatic transaminitis | 4 | 8 | 5 | – |
| Others | 1 | 2 | – | – |
| Total number of events | 49 | 53 | 27 | 6 |

(13.9%) experienced grade 4 toxicity. The main clinical toxicities were asthenia, fever, nausea, vomiting and psychiatric disorders (Table 2). All except six patients developed neutropenia, with grade 3 or 4 toxicity in 12 patients. Five patients had grade 3 hepatic cytotoxicity. Seventeen patients (47.2%) required either discontinuation or dose reduction because of toxicity.

### Consolidation phase

For the purpose of analysis, the maintenance phase was divided into four time periods: 1–3 months, 4–6 months, 7–9 months and 10–11 months (Tables 3 and 4). All patients were considered for toxicity.

The reasons for discontinuation of treatment were disease progression (14 patients) and toxicity (five patients) (Table 3). Another patient had a pregnancy after 9 months of treatment.

From months 1–3 of the consolidation treatment, 23/34 patients received 100% of the planned dose of IFN-α, while 17/31, 13/26 and 7/17 patients did so in the subsequent consolidation phases respectively (Table 1).

Seven patients had at least a grade 4 toxic event and nine at least grade 3 side-effects related to treatment during the overall consolidation phase. The most frequent severe clinical side-effects were asthenia and psychiatric disorders, while neutropenia and an increase in transaminases were the most frequent severe treatment-related biological changes (Table 4). Any side-effects were regularly reported irrespective of the period. Throughout the consolidation treatment, 19/34 patients (55.8%) who started the consolidation phase had either a dose reduction or discontinued therapy due to toxicity.

### End of treatment

Sixteen patients (44.4%) among the 36 initial patients completed the treatment after 12 months of IFN-α-2b.

### DISCUSSION

Owing to the results of the ECOG 1684 study (Kirkwood et al, 1996), IFN-α-2b is now licensed for use as adjuvant therapy in patients with a high-risk melanoma. Although this treatment cannot be considered as standard until the results are confirmed by another trial (ECOG 1690), we felt it was useful to test whether this treatment is feasible in terms of schedule, dose and toxicity outside the reference trial.

In comparison to the initial study of the ECOG, our results do not differ for the total given dose of IFN-α-2b, for toxicity or for the incidence of disease progression during treatment.

Whereas in the ECOG 1684 study (Kirkwood et al, 1996), 72.7% of patients received more than 80% of the theoretical dose, 66.6% did so in our study during the induction phase. Nevertheless, this period needs a thorough clinical and biological follow-up to check for the occurrence of grade 3 or 4 toxicity which was encountered by 50% of our patients and at least in 37% in the ECOG study (Kirkwood et al, 1996).

Significant toxicity was also seen in the consolidation phase, as 16 patients (47%) experienced a grade 3 or 4 toxicity, a similar figure to that in the ECOG study.

### CONCLUSION

Adjuvant treatment with high-dose IFN-α-2b for patients with malignant cutaneous melanoma, as reported by ECOG (Kirkwood

**Table 3** Feasibility of treatment

| Phase | | Induction 1 month | | Consolidation 1–3 months | | Consolidation 4–6 months | | Consolidation 7–9 months | | Consolidation 10–11 months | |
|---|---|---|---|---|---|---|---|---|---|---|---|
| Number of patients at start in a given period | 36 | | 34 | | 31 | | 26 | | 17 | | 16 |
| Stop treatment due to toxicity | | 1 | | 2 | | 0 | | 2 | | 0 | |
| Stop due to disease progression | | 1 | | 1 | | 5 | | 6 | | 1 | |
| Pregnancy | | | | | | | | 1 | | | |

 *© 1999 Cancer Research Campaign*

**Table 4** Toxicity by type and degree during the different periods of consolidation phase (WHO grade)

| | Period | 1 | 2 | 3 | 4 | Number of patients for period |
|---|---|---|---|---|---|---|
| Asthenia | 1–3 | 14 | 8 | 4 | 1 | 34 |
| | 4–6 | 12 | 9 | 4 | 2 | 31 |
| | 7–9 | 12 | 4 | 1 | 2 | 26 |
| | 10–11 | 9 | 3 | 2 | – | 17 |
| Fever | 1–3 | 5 | 1 | – | – | 34 |
| | 4–6 | 5 | 1 | – | – | 31 |
| | 7–9 | 2 | 1 | – | – | 26 |
| | 10–11 | – | 1 | – | – | 17 |
| Nausea/vomiting | 1–3 | 11 | 2 | – | – | 34 |
| | 4–6 | 11 | 1 | – | – | 31 |
| | 7–9 | 6 | – | – | – | 26 |
| | 10–11 | 7 | – | – | – | 17 |
| Psychiatric disorders | 1–3 | 2 | 5 | 1 | 1 | 34 |
| | 4–6 | 2 | 5 | 3 | – | 31 |
| | 7–9 | 1 | 7 | – | – | 26 |
| | 10–11 | 1 | 4 | – | 1 | 17 |
| Headache/myalgia | 1–3 | 1 | 2 | – | – | 34 |
| | 4–6 | – | 2 | – | – | 31 |
| | 7–9 | – | 2 | – | – | 26 |
| | 10–11 | – | 1 | – | – | 17 |
| Alopecia | 1–3 | 5 | 2 | 1 | – | 34 |
| | 4–6 | 8 | 1 | 1 | – | 31 |
| | 7–9 | 5 | 3 | – | – | 26 |
| | 10–11 | 4 | 2 | – | – | 17 |
| Neutropenia | 1–3 | 10 | 9 | 3 | 1 | 34 |
| | 4–6 | 5 | 15 | 5 | 1 | 31 |
| | 7–9 | 6 | 11 | 3 | 1 | 26 |
| | 10–11 | 4 | 5 | 4 | – | 17 |
| Hepatic transaminitis | 1–3 | 6 | – | 3 | – | 34 |
| | 4–6 | 6 | 2 | – | – | 31 |
| | 7–9 | 4 | 1 | – | – | 26 |
| | 10–11 | 1 | 2 | – | – | 17 |

et al, 1996), is feasible. Our results do not differ from those of the reference study in terms of schedule and the dose given of IFN-α-2b. Moreover, the significant toxicity reported by Kirkwood was also seen in our study.

### REFERENCES

Cole BF, Gelber RD, Kirkwood JM, Goldhirsch A, Barylak E and Borden E (1996) Quality-of-life adjusted survival analysis of interferon alpha-2b adjuvant treatment of high-risk cutaneous melanoma: an Eastern Cooperative Oncology Group study. *J Clin Oncol* **14**: 2666–2673

Kirkwood JM, Strawderman MH, Ernstoff MS, Smith TJ, Borden EC and Blum RH (1996) Interferon alpha-2b adjuvant therapy of high risk resected cutaneous melanoma: the Eastern Cooperative Oncology Group trial EST 1684. *J Clin Oncol* **14**: 7–17

*© 1999 Cancer Research Campaign*
